# Traversing myths and mountains: addressing socioeconomic inequities in the promotion of nutrition and physical activity behaviours

**DOI:** 10.1186/s12966-015-0303-4

**Published:** 2015-11-14

**Authors:** Kylie Ball

**Affiliations:** Deakin University, Centre for Physical Activity and Nutrition Research, 221 Burwood Hwy, Burwood, VIC 3125 Australia

**Keywords:** Socioeconomic disadvantage, Inequities, Inequalities, Interventions

## Abstract

**Background:**

In developed countries, individuals experiencing socioeconomic disadvantage – whether a low education level, low income, low-status occupation, or living in a socioeconomically disadvantaged neighbourhood – are less likely than those more advantaged to engage in eating and physical activity behaviours conducive to optimal health. These socioeconomic inequities in nutrition and physical activity (and some sedentary) behaviours are graded, persistent, and evident across multiple populations and studies. They are concerning in that they mirror socioeconomic inequities in obesity and in health outcomes. Yet there remains a dearth of evidence of the most effective means of addressing these inequities. People experiencing disadvantage face multiple challenges to healthy behaviours that can appear insurmountable. With increasing recognition of the role of underlying structural and societal factors as determinants of nutrition and physical activity behaviours and inequities in these behaviours, and the limited success of behaviour change approaches in addressing these inequities, we might wonder whether there remains a role for behavioural scientists to tackle these challenges.

**Discussion:**

This debate piece argues that behavioural scientists can play an important role in addressing socioeconomic inequities in nutrition, physical activity and sedentary behaviours, and that this will involve challenging myths and taking on new perspectives. There are successful models for doing so from which we can learn.

**Summary:**

Addressing socioeconomic inequities in eating, physical activity and sedentary behaviours is challenging. However, successful examples demonstrate that overcoming such challenges is possible, and provide guidance for doing so. Given the disproportionate burden of ill health carried by people experiencing socioeconomic disadvantage, all our nutrition and physical activity interventions, programs and policies should be designed to reach and positively impact these individuals at greatest need.

## Background

Eating a healthy diet, being regularly physically active, and not being too sedentary, are important actions that help to promote health, quality of life, functionality and longevity (e.g., [1, 2]). However, arguably the most important determinant of whether we lead a “healthy long life” is where we sit on the social scale within our society. The lower our socioeconomic position – our education level, occupational status, income, or the affluence of the neighbourhood in which we live - the worse our health and the more likely we are to die prematurely [3]. The increased risk of poor health is not limited to people living in absolute poverty or extreme disadvantage; rather, we see a social gradient such that even people in the middle of the socioeconomic ladder have poorer health than those at the top.

There is extensive evidence of the socioeconomic gradient throughout the world. It is of such concern, that the World Health Organisation Commission established in 2005 to help address health inequities, concluded that ‘social injustice is killing on a grand scale’ [4]. To consider just a few examples, a child born in an affluent neighbourhood of Glasgow, Scotland can expect a life up to 15 years longer than a child living only a few kilometres away [5]. Indigenous Australians, who are amongst the most socioeconomically disadvantaged groups in any developed society, will on average live 10 years less than non-Indigenous Australians [6]. Within the US, there is a 9 year difference in life expectancy between those with higher and lower levels of education [7]. These are shocking differences in life expectancy. But people experiencing socioeconomic disadvantage don’t only die sooner, they will also spend more of their shorter lives with a disability or illness.

Can behavioural scientists play a role in helping to address socioeconomic inequities in health behaviours and outcomes? In considering this question, it is important to note that health inequities are not explained by behaviours alone. Health inequities result from social inequities – that is, social determinants such as employment conditions, living standards, housing, and income [3]. Addressing health inequities requires addressing social inequities – for example, via progressive social and economic policies that address inequities in education, housing, employment, income and access to healthcare. A focus on behaviours doesn’t and shouldn’t replace this, but it is part of the solution. Physical activity and healthy eating are key health protective factors and inequities in these behaviours play a role in contributing to health inequities. This is recognized in the Marmot Review [3], where one of the six policy objectives recommended for reducing health inequities is to “strengthen the role and impact of ill health prevention”, which emphasises the need to prioritise investment in health promotion aimed at changing individual behaviours including diet and physical activity behaviours.

In attempting to improve physical activity and eating behaviours, behavioural scientists often take an individually-focused perspective. Without taking adequate account of broader social determinants, such approaches run the risk of failing to benefit those most at need, or even of widening inequities, if they are more effective amongst those most socioeconomically advantaged. Indeed, many physical activity or eating behaviour change interventions reported in the literature have had little impact (either because of poor reach, or lesser effectiveness) among people who are socioeconomically disadvantaged [8, 9]. However, behavioural scientists can play a key role in contributing to the reduction of inequities via the implementation of behaviour change interventions for those who are socioeconomically disadvantaged, if these are designed effectively, and in a manner that addresses a number of key challenges. These include consideration of the underlying causes of inequities; ensuring interventions are tailored to the needs and capacities of the target population; and reaching and engaging these populations to implement interventions in deprived settings. This paper provides selected examples of interventions that have addressed these issues.

### Evidence for inequities in diet, physical activity and sedentary behaviours

Inequities in diet and physical activity are observed across multiple indicators and populations. For example, a review of European studies [10] found that socioeconomic disadvantage was associated with less favourable dietary behaviours among adults. Other reviews show that obesity-related dietary behaviours were more common among children whose parents had a lower education level [11], and among adults who lived in a socioeconomically disadvantaged neighbourhood [12]. Socioeconomic position consistently predicts participation in leisure-time physical activity including sport among adults [13], although the evidence is slightly less consistent among children and adolescents [14], possibly suggesting that socioeconomic gradients in physical activity don’t emerge until later in life – for instance, once the impact of school-based physical education and sport are removed. The literature pertaining to sedentary behaviours is more recent but there is evidence from several reviews showing that education is inversely associated with TV viewing time among adults, and that children from socioeconomically disadvantaged backgrounds spend more time in sedentary screen behaviours [15, 16].

### What causes these inequities?

According to a social ecological model [17] there are a number of determinants of diet, physical activity and sedentary behaviour across four key domains (individual, interpersonal, community, society). Any of these might plausibly vary according to socioeconomic characteristics. In considering initiatives to address inequities in behaviours, we need to avoid approaches that place all of the onus on individuals to change, as models such as the social ecological model emphasise that these are unlikely to be effective without considering upstream social determinants. However, we also need to take care not to overlook a focus on individual and their behaviours. Models like this are valuable in that they highlight the importance of all levels of influence, including the individual level. For example, we would be unlikely to advocate for cutting out smoking cessation or alcohol reduction programs because these place too much onus on individuals. For those facing greater disadvantage, behaviour change approaches may need to be implemented differently, or with greater intensity, and ideally they will be supplemented with action at community and societal levels, but behaviour change approaches are part of the solution.

#### Traversing mountains

A participant in one of our trials, who was struggling with adopting healthier eating behaviours, suggested that sticking to a healthy eating plan was as hard as climbing a mountain. This analogy is not difficult to relate to. The average adult in Australia is overweight, habitually inactive, and eats very few vegetables [18]. For people in these circumstances, trying to adopt and stick to a healthy eating or physical activity plan can seem as challenging as starting off on a mountain climb. The ease and success with which this behaviour change mountain is traversed is variable, and dependent on individual perspectives and circumstances.

From one perspective, traversing this mountain may be relatively straightforward. The starting point may not be at the very bottom; others may have walked here before, or may be traversing the mountain now, and can provide support and role modelling; the route may not be particularly taxing, in terms of required time, energy, planning/skills, or equipment and resources; and the terrain may be walkable, safe, and aesthetically pleasant. Alternatively, an individual might come to this challenge of changing long-established behaviours with no prior experience that can help traverse it, or worse, with very negative experiences from early in life. Socioeconomic disadvantage begins to exert influences early in life, with effects extending into adulthood. For example, children from socioeconomically disadvantaged families are less often exposed to healthy foods from early in life [19], which can shape taste preferences and habits throughout life. Socioeconomically disadvantaged individuals are also more likely to have had less exposure to positive physical activity experiences from early in life [20], which may help to explain the socioeconomic gradients observed in self-efficacy for physical activity, a key determinant of physical activity behaviour [21].

There is also evidence demonstrating socioeconomic gradients in social support and social norms related to healthy eating and physical activity behaviours [20–22], with individuals from disadvantaged backgrounds receiving the least support. Low status occupations can also pose additional challenges. Hours can be long and inflexible; conditions can be challenging; work resources and benefits are often low. Job control over things like skill use, time allocation and organisational decisions is also often low [23]. Women in particular are over-represented among the working poor, and are at heightened risk of experiencing stresses associated with juggling domestic responsibilities with low paid, part-time work [24]. Such circumstances leave little energy for planning, shopping for and preparing healthy meals; or for physical activity [20].

Another aspect of socioeconomic disadvantage that has recently received attention concerns the potential impact of scarcity on cognition. Mullainathan & Shafir [25] propose that the circumstances of being poor, and all of the related concerns associated with that, require so much mental energy that people have less remaining brainpower to devote to other areas of life. They describe this as a “cognitive tax” on mental bandwidth, a tax which distorts decision-making, leading to impaired cognitive function. This has implications given the planning that is required to make healthy food purchasing decisions on a limited budget; or to build in physical activity to a schedule with little if any leisure time.

Financial barriers also pose significant challenges to achieving a healthy diet amongst those who are socioeconomically disadvantaged. Australian data, for example, show that the average costs of a healthy weekly meal plan required only 20 % of the income of an average income family, but double that for a welfare-dependent family; and a large family in the lowest income quintile would need to spend 56 % of their weekly income on food [26, 27]. In other words, a healthy diet is not a realistic choice for some families.

Neighbourhoods that are disadvantaged are also often not conducive to physical activity or healthy eating. Access to stores selling healthy food is lacking in some disadvantaged neighbourhoods, particularly in the US, although evidence elsewhere is less consistent [28]. Many people living in disadvantaged neighbourhoods report that their neighbourhoods are not aesthetically pleasant, or they do not have access to recreational facilities that support and make physical activity feasible and enjoyable [20, 21, 29]. Some disadvantaged areas have far more serious problems. In the US, young people in disadvantaged neighbourhoods witness more severe violence – in some studies, up to a quarter of young people in low-income urban neighbourhoods had witnessed a murder [30]. Against that backdrop it is not difficult to see why health promoting behaviours are not the highest priority for many young people and their families.

Collectively this evidence suggests that people experiencing socioeconomic disadvantage often face extraordinary challenges in adopting and maintaining healthy eating or physical activity behaviours. Their reasons for not doing so are real and can appear insurmountable. What might at first glance appear to be the ‘same’ task can be experienced vastly differently and we need to take an approach to behaviour change that incorporates these perspectives. In order to look beyond our own ‘advantaged scientist’ perspective to better understand the needs and insights of disadvantaged groups, we may need to start by confronting some commonly espoused myths.

### Challenging myths

One common perspective is that people who are disadvantaged are not interested in improving their health/behaviours, and are hence inevitably “hard to reach/engage”. Considering the substantial challenges to behaviour change experienced by those who are socioeconomically disadvantaged, as outlined in the preceding section, this perspective is perhaps well-founded. Indeed there is evidence that participants from disadvantaged backgrounds are often under-represented in physical activity and nutrition interventions [9, 31]. However, this is not inevitable. One example, the experience of a nutrition promotion study in the supermarket setting, shows that it is not necessarily difficult to engage participants from disadvantaged groups. ShopSmart 4 Health was a randomised controlled trial of a behaviour change program aimed at helping to enhance the confidence and skills of low-income women in budgeting for, purchasing, and preparing fruit and vegetables inexpensively [32]. The study recruited via a large Australian supermarket chain using their store loyalty program. We identified cardholding women from disadvantaged areas and initially mailed out 1000 recruitment flyers. The intervention group received behaviour change activities and resources including newsletters, budgeting activities and costed recipes; they also had the opportunity to participate in a supermarket tour, with other participants and with a dietitian. All participants received loyalty points, equivalent to about $15 in total, and also a $20 shopping voucher for completing surveys. Contrary to the perspective that people experiencing disadvantage are not interested in health promotion, or are inevitably hard to reach, the response to the ShopSmart was so high that the recruitment target was met within 4 weeks, with 248 eligible women recruited after our first mailout, and consequently additional planned recruitment cancelled. Similarly, retention rates were extremely high (98 % by the conclusion of the 6-month intervention, and 95 % 12 months post-intervention).

Why did ShopSmart work to attract and retain low-income participants? Several factors may have contributed. The program was embedded into existing settings - the supermarkets where women were already shopping, and backed onto a loyalty scheme in which they were already engaged, so it didn’t require any ‘additional’ efforts. It also addressed needs identified by the target group in preliminary work and pilot testing. These were women who were struggling to feed themselves and their families on limited funds, and they needed quick, appealing, cheap ideas, which is what the intervention provided. It included a social support element – the shopping tours – which were rated favourably by those who attended. Possibly the women were also attracted by the incentives provided – while of small monetary value, the shopping vouchers and the loyalty points at the supermarkets where women already shopped, were valued. Our findings suggest that there does appear to be a high degree of interest among low-income groups in nutrition promotion strategies and if offered the right program, these groups are not inevitably hard to reach.

Another myth is that when designing interventions we should look to the general evidence base and apply what has been shown to work. Two of the common characteristics of behaviour change intervention approaches that are effective in the general population are more extensive use of a theoretical framework, and the use of multiple behaviour change techniques. Common sense then might tell us to apply this established evidence to working with socioeconomically disadvantaged groups. However, there is some literature suggesting otherwise. In their review of behaviour change interventions among low-income groups, Michie and colleagues [33] found that there was “No obvious association between reported use of theory, and whether or not the intervention was effective”. They also found that interventions that were effective tended to use fewer behaviour change techniques than did ineffective interventions. In another review and meta-analysis, we examined elements of effective interventions to promote physical activity among socioeconomically disadvantaged women [34]. We also found that effective interventions could not be distinguished from ineffective interventions on the basis of either the use of a theoretical framework, or the number of behaviour change techniques used. The main factor that did distinguish effective interventions was the use of a social or a group component - whether it was group education meetings, group practical sessions or both.

Where does this leave us in terms of our established wisdom regarding theory and behaviour change strategies? Possibly, our existing behaviour change approaches and evidence from the general population may not be fit for purpose when we’re working with disadvantaged populations. One consistent finding of reviews of nutrition and physical activity interventions that focus specifically on socioeconomically disadvantaged populations is that there is a lack of evidence on the effectiveness of interventions in these groups [34–36]. Hence rather than discount existing theoretical models, more sensitive models based on additional evidence about the most effective ways of intervening with those who are disadvantaged are required. This may require consideration, application and evaluation of modified theoretical models that, for example, incorporate a stronger role for challenging contextual factors (e.g., the impact of scarcity) likely to be particularly pertinent amongst those who are socioeconomically disadvantaged.

## Discussion

Despite the many barriers to behaviour change faced by those experiencing socioeconomic disadvantage, several initiatives demonstrate that it is possible to promote healthy eating or physical activity in disadvantaged groups. For example the workplace POWER (Preventing obesity without eating like a rabbit) intervention was a successful behaviour change weight loss intervention targeting men who were overweight and employed as blue collar shift workers [37]. That 12-week intervention achieved good recruitment and retention rates, and positive effects on outcomes including physical activity, sweetened beverage intakes and body weight. The intervention achieved this by understanding and catering to the key motivators of the target group – for example, assuring participants that they wouldn’t need to ‘eat like a rabbit’, and could still consume in moderation foods and drinks they enjoyed, including beer. The intervention was also embedded in an existing setting, the workplace; and incorporated social support elements including management support and group-based competitions.

Heart Foundation Walking is another example of an initiative that has successfully engaged with socioeconomically disadvantaged groups. Heart Foundation Walking is the largest walking group program in Australia. It represents a universal approach to physical activity promotion, targeting the whole population, including but not limited to disadvantaged participants. It is a free community-based program, so addresses economic barriers faced by some disadvantaged groups. The program involves the Heart Foundation partnering with local coordinators to establish walking groups led by volunteers, people who live in the communities and lead and participate in the walks. The approach is flexible; the groups can be any size, and walk at various times, days, lengths and levels of difficulty. There is also a virtual walking community, which caters for those who either can’t access the groups due to distance or other barriers, or who simply prefer to walk on their own but have the support of an online community. The groups cater for a range of specialty groups with different needs, including Aboriginal and Torres Strait Islander people, and people from culturally and linguistically diverse backgrounds.

Data from the program’s 2012 evaluation show that over the last 20 years, more than 75,000 people have participated in the program, collectively participating in more than 3 million walks. Currently there are more than 20,000 registered walkers in 1307 walking groups across Australia. The average group walks for 49 min once/week. Retention rates are extremely high, with 96 % of groups and 92 % of participants still active at six months, and the average group active for 3.6 years, with some groups walking for over 20 years. A further major success of the approach is its engagement of people experiencing socioeconomic disadvantage. Fifty-six percent of participants in the group’s 2012 evaluation had a household income below the Australian median of around $70,000, and nearly a quarter had a household income of less than $25,000, a very low income threshold. What does the program offer that caters so well to disadvantaged groups? In one survey of more than 3500 walkers, the main motivating factor for people to continue walking with a group was for social reasons, endorsed by 56 % of walkers. Health was the third most important reason, endorsed by only a quarter of walkers.

Finally, SHOP@RIC is a nutrition promotion intervention trial undertaken in remote Indigenous communities. Indigenous Australians are highly disadvantaged in terms of socioeconomic characteristics, and also diet and health [38]. Indigenous people living in remote communities in Australia have the poorest health outcomes of any population group in high income countries, and poor nutrition, particularly low fruit and vegetable intakes, contributes substantially to this [39]. One of the key challenges to addressing these problems, a challenge voiced by the residents of remote communities [40], is the high cost of fresh foods. For example, a healthy food basket costs 53 % more in remote communities in the Northern Territory of Australia compared to the capital city [41]. Hence the need for a study like SHOP@RIC came directly from the communities, and this study addressed this need by testing the impact of a 20 % price discount only and a combined price discount and in-store nutrition education strategy on purchasing of fruit & vegetables, diet drinks & water in 20 remote Aboriginal communities [42]. By incorporating strategies aimed at both individual behaviour change and improving food accessibility (through pricing strategies), SHOP@RIC addressed two of the key domains (consumer and food environments) suggested as priorities by key policy frameworks such as NOURISHING [43].

The price discount was applied in stores in the 20 communities, and was promoted through posters at the front of stores, shelf labels and price ticketing on targeted products. The nutrition education component was developed using an intervention mapping approach including a needs assessment, by a working group with public health nutrition, health promotion and retail expertise and extensive remote Indigenous community experience. It included posters, activity sheets, cooking demonstrations, taste-testings and receipt reward prize draws that promoted fruit, vegetable and water consumption. As well as having strong buy-in from the public health nutritionists and the store managers in the communities, a key element was the involvement of community coordinators. These were local people, at least one in each community, who were trained to assist with implementation of the nutrition education strategy. These strategies helped to ensure that it was meaningful, engaging and culturally appropriate for Indigenous people in remote communities.

While the main outcomes are under analysis, feedback and early results are promising. The approach was well received, by the residents themselves, and by the various stakeholders, including community coordinators and store managers, all of whom indicated they wanted more of this type of intervention implemented over the longer term. Early results suggest some positive impacts of some intervention components on key dietary indicators.

Several key shared elements likely contributed to the success of these two initiatives, Heart Foundation Walking and SHOP@RIC. Both directly addressed the community needs identified by the communities – the social aspects of walking, or the high costs of healthy eating in remote communities. They were co-led by local community members; they had good buy in & leadership from the communities and fostered community capacity through ongoing training and support amongst the walk volunteers and community coordinators for example. They addressed the key barriers, including economic barriers. They also both incorporated a social element, through the social aspects of walking, or the use of a central gathering point, the store in remote communities, as the intervention setting. Both were based on partnerships and participatory decision-making between the lead organisation and the communities that helped to address the key needs of those involved.

## Conclusions

The literature shows relatively consistent evidence of socioeconomic gradients in diet, leisure time physical activity, and some markers of sedentary behaviour. People experiencing socioeconomic disadvantage face a range of challenges that can substantially hinder efforts to adopt healthy eating and physical activity behaviours. While there are several examples of engaging socioeconomically disadvantaged groups in promoting nutrition and physical activity behaviours, evidence of best practice in this field remains scarce. To address these social injustices, as behavioural scientists, policymakers and practitioners, we should all be designing our interventions to reach and impact those with greatest need, and asking not only do our policies and programs work to improve nutrition, physical activity or sedentary behaviours; but also do they work to reduce inequities in these behaviours.
